# Anti-*Toxoplasma gondii* antibodies in patients with beta-hemoglobinopathies: the first report in the Americas

**DOI:** 10.1186/s13104-017-2535-7

**Published:** 2017-06-14

**Authors:** Marina Neves Ferreira, Claudia Regina Bonini-Domingos, Isabeth Fonseca Estevão, Clarice Lopes de Castro Lobo, Gisele Cristina Souza Carrocini, Aparecida Perpétuo Silveira-Carvalho, Octávio Ricci, Luiz Carlos de Mattos, Cinara Cássia Brandão de Mattos

**Affiliations:** 1 Department of Biology, Universidade Estadual Paulista “Júlio de Mesquita Filho”, Instituto de Biociências, Letras e Ciências Exatas-IBILCE-UNESP, São José do Rio Preto, SP Brazil; 2 Immunogenetics Laboratory, Department of Molecular Biology, Faculdade de Medicina de São José do Rio Preto-FAMERP, Avenida Brigadeiro Faria Lima, 5416, São José do Rio Preto, SP 15090–000 Brazil; 3 FAMERP Toxoplasma Research Group, Faculdade de Medicina de São José do Rio Preto, São José do Rio Preto, SP Brazil; 4 Clinical Hematology Division, Instituto de Hematologia Arthur de Siqueira Cavalcanti-HEMORIO, Rio de Janeiro, RJ Brazil; 5 Department of Medicine, Faculdade de Medicina de São José do Rio Preto-FAMERP, São José do Rio Preto, SP Brazil; 6 Regional Blood Center-Hemocentro de São José do Rio Preto-Fundação Faculdade Regional de Medicina-FUNFARME, São José do Rio Preto, SP Brazil; 7 Faculdade de Medicina Veterinária e Zootecnia, Universidade de São Paulo, São Paulo, Brazil

**Keywords:** Beta hemoglobinopathies, *Toxoplasma gondii*, Toxoplasmosis, Sickle cell disease, Beta-thalassemia

## Abstract

**Background:**

In Brazil, there have been no previous studies of *Toxoplasma gondii* infection in sickle cell anemia patients and carriers of severe forms of beta-thalassemia. This study evaluated *T. gondii* infection in patients with beta-hemoglobinopathies.

**Methods:**

A total of 158 samples, 77 (48.7%) men and 81 (51.3%) women, were evaluated. Three groups were formed: G1 (85 patients with sickle cell disease); G2 (11 patients with homozygous beta-thalassemia; G3 (62 patients with heterozygous beta-thalassemia). ELISA was employed to identify anti-*T. gondii* IgM and IgG antibodies, and molecular analysis was performed to determine beta-hemoglobin mutations. Fisher’s exact test was used to compare frequencies of anti-*T. gondii* IgM and IgG antibodies in respect to gender and age.

**Results:**

Anti-*T. gondii* IgG antibodies were found in 43.5% of individuals in G1, 18.1% in G2 and 50% in G3. All samples from G1 and G2 were seronegative for anti-*T. gondii* IgM antibodies, but 3.2% from G3 were seropositive. Considering anti-*T. gondii* IgG antibodies, no statistical significant differences were found between these groups nor in seroprevalence between genders within each group. Despite this, comparisons of the mean ages between G1, G2 and G3 were statistically significant (G2 vs. G1: p value = 0.0001; G3 vs. G1: p-value <0.0001; G3 vs. G2: p-value = 0.0001).

**Conclusion:**

A comparison by age of patients with sickle cell anemia showed a trend of lower risk of infection among younger individuals. Therefore, this study demonstrates that *T. gondii* infection occurs in patients with beta-thalassemia and sickle cell anemia in Brazil as seen by the presence of anti-*T. gondii* IgM and IgG antibodies.

## Background

Sickle cell disease is a hereditary hemoglobinopathy characterized by the presence of hemoglobin (Hb) S, generated from a point mutation in the β-globin gene. Homozygosity for this point mutation leads to severe anemia with clinical manifestations such as hemolytic anemia, pain crises, infections, ulcers and recurrent vaso-occlusive events [1, 2].

In Brazil, this is the most common hereditary disease due to the influence of African descendants in the composition of the population and, in particular, to the high rate of miscegenation [3, 4]. Transfusions of red blood cells are very important to sickle cell disease patients, as this can reduce or even prevent acute and chronic complications.

Beta-thalassemia is the most common autosomal recessive inheritance disorder worldwide. The variability in the disease phenotypes ranges from clinically asymptomatic to severe anemia [5, 6].

Despite the benefits that blood transfusions offer to patients with beta-thalassemia, some risks should be considered such as the high transmission risk of infectious agents (viruses, bacteria and parasites). In fact, cases of malaria and Chagas disease have been identified after blood transfusions [7–10]. Infections, mainly septicemia and pneumonia, account for 45% of deaths in sickle cell disease children, even though these infections are not necessarily acquired by transfusions [11, 12].

Toxoplasmosis is a globally prevalent zoonotic disease caused by the obligate intracellular parasite *Toxoplasma gondii* (Phylum Apicomplexa), which mainly infects birds and mammals, including man. Transmission is primarily by eating raw or undercooked meat containing cysts of the parasite and consuming food and water contaminated with oocysts. However, there are other ways to get infection, such as through transplants and blood transfusions, as well as congenital transmission [13–16].

Siegel et al. [17] described one of the first instances of *T. gondii* transmission by the transfusion of blood products in which four immunocompromised individuals developed toxoplasmosis. Screening for anti-*T. gondii* in donated blood is not compulsory in many countries, including Brazil, but studies have shown the importance of this transmission route [18, 19]. Despite the inherent risk, there are many questions about the effectiveness of implementing testing to identify toxoplasmosis [20].

Karakaş et al. [21], in a study conducted with beta thalassemia major patients in Aydin Province, Turkey, investigated the possible relationship between *T. gondii* infection and blood transfusions that these patients received. They found that seropositivity for anti-*T. gondii* antibodies of the analyzed group was greater than a control group, but no statistical significance was verified. Given the fact that no previous studies have investigated *T. gondii* infection in patients with beta-thalassemia and sickle cell anemia in Brazil, the aim of this study was to evaluate the presence of anti-*T. gondii* IgM and IgG antibodies in patients with beta-thalassemia major and intermedia and with sickle cell anemia diagnosed and treated in three referral centers.

## Methods

### Ethical aspects of the study

This study was approved by the Ethics Committees of the Universidade Estadual Paulista “Júlio de Mesquita Filho”, Instituto de Biociências, Letras e Ciências Exatas (UNESP/IBILCE), and the Faculdade de Medicina in São José do Rio Preto (FAMERP) and the Instituto de Hematologia Arthur de Siqueira Cavalcanti, Rio de Janeiro (HEMORIO). Individuals who agreed to participate were informed about the nature of the study and all subjects and the patents or legal guardians of under 18-years-old signed informed consent forms.

### Patients

This study enrolled 158 patients attended at three referral centers for the treatment of beta hemoglobinopathies in southeastern Brazil (the Hemocentro de São José do Rio Preto, SP, the Instituto Estadual de Hematologia Arthur de Siqueira Cavalcanti-HEMORIO, Rio de Janeiro, RJ and a private clinic in the city São Carlos, SP). All patients signed a consent form allowing their participation on this study.

The patients were allocated to three groups: G1 (85 patients with sickle cell disease); G2 (11 patients with homozygous beta-thalassemia, two clinically classified as beta-thalassemia intermedia and nine as beta-thalassemia major) and G3 (62 patients with heterozygous beta-thalassemia).

Carriers of sickle cell anemia and beta thalassemia major receive regular blood transfusions because this is the most appropriate and effective treatment. Otherwise, the rate of transfusions for thalassemia intermedia carriers is variable and may not be regular. In general, patients with sickle cell anemia receive blood transfusions when the hemoglobin level is 6 g/dL or lower. All sickle cell anemia patients were on regular treatment using hydroxyurea and the disease was controlled.

The nine beta thalassemia major patients were receiving transfusions every 15 or 21 days, but the two beta thalassemia intermedia patients were not receiving regular blood transfusions. None of these patients was using hydroxyurea.

Diagnosis of these hematological disorders occurs soon after birth or within the first few years of life due the severe symptoms.

The 62 individuals with heterozygous beta thalassemia are asymptomatic and did not receive blood transfusions. They were analyzed to compare the prevalence of *T. gondii* infection in individuals that received regular transfusions and in those that did not.

**Fig. 1:**
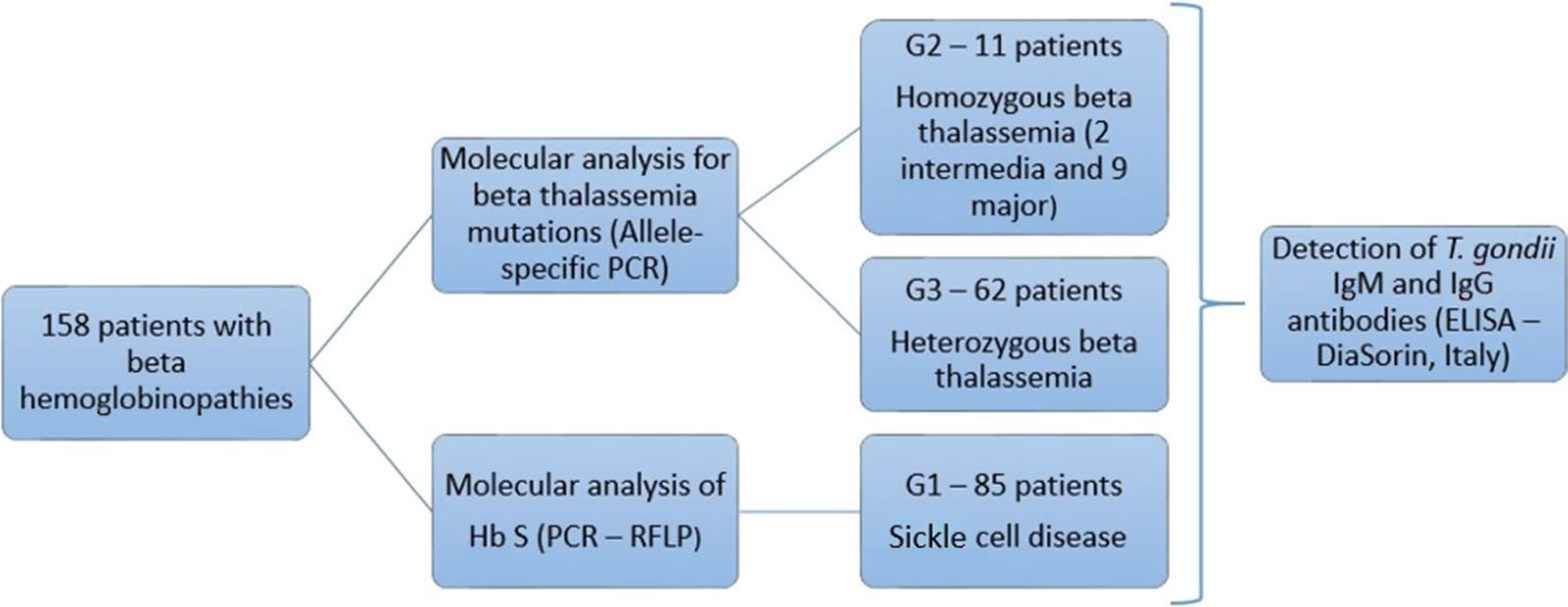
Flow diagram showing the methodology that were conducted in the analyzed samples, for diagnosis of hematological disorders and infection of *T. gondii*

Figure 1 shows the realized steps to diagnose hematological disorders and *T. gondii* infection in this research.

### Blood sampling

Blood samples were collected in tubes with ethylenediaminetetraacetic acid (EDTA). An investigation of *T. gondii* infection (IgM and IgG antibodies) was made and DNA was extracted from leukocytes.

### Detection of anti-*T. gondii* IgM and IgG antibodies

Anti-*T. gondii* IgM and IgG antibodies were investigated using the enzyme-linked immunosorbent assay (ELISA) test with the ETI-TOXOK (IgM) and ETI-TOXOK (IgG) commercial kits (DiaSorin, Saluggia, Italy) according to manufacturer’s instructions.

### Molecular diagnosis of beta-thalassemia

Genomic DNA was extracted from peripheral blood samples and DNA purity was determined by the ratio of optical density (OD) at 260 and 280 nm in a NanoDrop ND100 spectrophotometer (Thermo Scientific, USA). DNA samples were diluted to a final concentration 100 ng/μL.

### Molecular analysis of Hb S by polymerase chain reaction-restriction fragment length polymorphism (PCR–RFLP)

Detection of the Hb S mutation was by PCR–RFLP [22]. The primers used for the amplification of codon 6 were the P 277 primer (sense): 5′ GGC AGA GCC ATC TAT TGC TTA 3′ and the P 278 primer (antisense): 5′ ACC TTA GGG TTG CCC ATA AC 3′. After amplification, the 382 bp fragment was digested by the FastDigest *Dde*I restriction enzyme at 37 °C for 5 mins. Hb S eliminates the restriction site for the *Dde*I enzyme, so fragments of 6, 88 and 288 bp are produced (the first one is not seen in agarose gel). Fragments of 6, 87, 88 and 201 bp are identified in the absence of this mutation.

### Molecular analysis of beta-thalassemia mutations by allele-specific PCR

Because high frequencies of some mutations of beta-thalassemia are found in the Brazilian population [23, 24], the following were investigated: CD39 (HBB: c.118C>T), IVSI-110 (HBB: c.93-21G>A), IVSI-6 (HBB: c.92 + 6T>C) and IVSI-1 (HBB: c.92 + 1G>A). Detection of these mutations was performed by allele-specific PCR, using the PS39 W (5′ GAC TCA AAG AAC CTC TG 3′) and PS39M primers (5′ GAC TCA AAG AAC CTC TA 3′) for the CD39 mutation; the TB110W (5′ GGG TGG GAA AAT AGA CC 3′) and TB110M primers (5′ GGG TGG GAA AAT AGA CT 3′) for the IVSI-110 mutation; the IVSI6W (5′ GTC TTG TAA CCT TGA TA 3′) and IVSI6M primers (5′ GTC TTG TAA CCT TGA TG 3′) for the IVSI-6 mutation and the IVSI1W (5′ GTG ACC TTG ATA CCA AC 3′) and IVSI1M primers (5′ GTG ACC TTG ATA CCA AA 3′) for IVSI-1 mutation. [25].

### Statistical analysis

The results were compared using the GraphPad statistics program (version 3.1). The T-test was used to compare mean ages. Fisher’s exact test was used to compare frequencies of anti-*T. gondii* IgM and IgG antibodies in respect to gender and age. Moreover, Odds Ratio and the 95% confidence interval were calculated. Statistical significance was set for an alpha error of 5% (p-value <0.05).

## Results

A total of 158 blood samples—from 77 (48.7%) men and 81 (51.3%) women—were tested. The distribution of gender in each group is shown in Table 1.

**Table 1:** Stratification by gender in each of the analyzed groups

	Male, n (%)	Female, n (%)	Total, n
G1	39 (45.8)	46 (54.1)	85
G2	8 (72.7)	3 (27.3)	11
G3	30 (48.3)	32 (51.61)	62

*G1* sickle cell disease patients, *G2* homozygous beta-thalassemia patients, *G3* heterozygous beta-thalassemia minor patients

The mean ages were 29.7 ± 15.0, 27.4 ± 18.0 and 51.2 ± 18.0 years for groups G1, G2 and G3, respectively. The mean age of the heterozygous beta-thalassemia carriers (G3) was higher than the sickle cell anemia (G1) and homozygous beta-thalassemia patients (G2). Comparisons of the mean ages between groups were statistically significant (G2 vs. G1: p-value = 0.0001; G3 vs. G1: p-value <0.0001; G3 vs. G2: p-value = 0.0001).

All samples from G1 and G2 were seronegative for anti-*T. gondii* IgM antibodies, thus, comparisons between groups were made considering only results for anti-*T. gondii* IgG antibodies (Table 2). No statistical significant differences were found between groups with regard to positive and negative serology for anti-*T. gondii* IgG antibodies. Moreover, there were no statistically significant differences in seroprevalence for anti-*T. gondii* antibodies between genders within each group.

**Table 2:** Frequencies of seropositive and seronegative samples for anti-*T. gondii* antibodies in the analyzed groups as determined by ELISA

	IgM				IgG			
	Seropositive		Seronegative		Seropositive		Seronegative	
	n	%	n	%	n	%	n	%
G1 (n = 85)	0		85	100	37	43.5	48	56.5
G2 (n = 11)	0		11	100	2	18.1	9	81.9
G3 (n = 62)	2	3.2	60	96.8	31	50	31	50

G1 vs. G2 → p-value = 0.1904; OR = 3.47; 95% CI = 0.70–17.03

G1 vs. G3 → p-value = 0.5038; OR = 0.77; 95% CI = 0.40–1.49

G2 vs. G3 → p-value = 0.0972; OR = 0.22; 95% CI = 0.04–1.11

*G1* sickle cell disease patients, *G2* homozygous beta-thalassemia patients, *G3* heterozygous beta-thalassemia minor patients

For further analyses, the individuals of each group were classified as pediatric or adult patients; under 18 year olds were categorized as pediatric patients. The seroprevalence for anti-*T. gondii* antibodies of these subgroups was analyzed in Table 3. The age comparison of patients with sickle cell anemia indicates that there is a trend of lower risk of infection among younger individuals (pediatric patients). This condition was not observed in beta-thalassemia patients because there were no under 18-year-old patients in G3 and the number of homozygous beta-thalassemia individuals infected (G2) was very small.

**Table 3:** Frequency of samples reactive for anti-*T. gondii* IgG antibodies stratified according to age of individuals

	Seropositive, n (%)	Seronegative, n (%)	
G1			
≤17 years	5 (25.0)	15 (75.0)	p-value = 0.072 OR = 0.34 95% CI = 0.11–1.06
≥18 years	32 (49.0)	33 (51.0)	
G2			
≤17 years	0	5 (100.0)	p-value = 0.45 OR = 0.16 95% CI = 0.006–4.36
≥18 years	2 (33.0)	4 (67.0)	

*G1* sickle cell disease patients, *G2* homozygous beta-thalassemia patients

## Discussion

The frequency of *T. gondii* infection is high in the states of São Paulo and Rio de Janeiro, especially when considering the population of pregnant women and patients with ocular toxoplasmosis (Table 4). The same is true for the population of this study, which includes, for the first time in Brazil, individuals with beta hemoglobinopathies.

**Table 4:** Frequency of *T. gondii* infection in different populations from Rio de Janeiro and São Paulo state, Brazil

City	%	N	Population	Age range (years)	Reference
São Paulo State					
Botucatu	60.0	913	Pregnant women	No information	[36]
Presidente Prudente	33.8	80	Students	18–35	[37]
São José do Rio Preto	64.1	1006	Pregnant women	12–44	[38]
São José do Rio Preto	74.5	349	Ophthalmology	18–88	[39]
São José do Rio Preto	54	312	Blood donors	27.5 ± 6.9	[40]
São Paulo	22	262	Ophthalmology	4–88	[41]
Araraquara	58	233	Pregnant women	18–20	[42]
São Carlos	50	62	Beta-thalassemia heterozygous	24–92	This study
Rio de Janeiro State					
Campos dos Goytacazes	90.0	110	Ophthalmology	6–35	[43]
Santa Rita de Cássia, Barra Mansa	65.9	1071	Ophthalmology	6 months–88	[44]
Rio de Janeiro	21.8	839	Students	20–30	[45]
Miracema	75.1	832	Pregnant women	No information	[46]
Rio de Janeiro	18.1	11	Beta-thalassemia homozygous	7–59	This study
Rio de Janeiro	43.5	85	Sickle cell anemia	6–63	This study

Most sickle cell anemia patients and those with severe forms of beta-thalassemia depend on transfusions of blood products to improve the clinical condition resulting from these hemoglobinopathies [7, 26–29]. However, there is a risk of infections by viruses, bacteria and parasites with transfusions once serological screening is not compulsory for all microorganisms [10, 17, 18, 30–32]. Despite the lack of data on blood transfusions performed in the individuals analyzed in the current study, we should consider this risk, and more detailed studies should be performed to verify the possible transmission of pathological agents in blood transfusions.

This study did not find differences between the groups considering the serology for anti-*T. gondii* IgG antibodies. Similar to these results, Karakaş et al. [21] found no difference between a control group and a group of patients with beta-thalassemia major in respect to serology for these antibodies. This suggests that the treatment of patients with severe hemoglobinopathies requiring frequent red blood cell transfusions does not appear to influence the rate of infections with *T. gondii*. However, it should be considered that the groups compared in this study do not have mean age equivalent, and the patients present in each group come from different geographical area. Despite these inequalities, this study alone is inconclusive about any relation between patients with beta hemoglobinopathies and infection with *T. gondii*.

As no statistically significant difference in the frequencies of *T. gondii* infection was observed between groups, it is possible that all patients were infected mainly by natural means (water and food containing oocysts), and that the transfusion of red blood cells does not constitute a risk factor for infection.

In this study, the differences in mean ages between groups may be explained by the fact that the carriers of these two serious diseases (sickle cell anemia and homozygous beta-thalassemia) are referred to treatment centers very early in their lives [11, 12, 27, 29, 33–35]. Thus, the samples are from young patients who are under treatment and consequently these two groups had lower mean ages compared to the group of patients with heterozygous beta-thalassemia.

When comparing the sickle cell anemia patients by age group (pediatric vs. adult patients), there is a tendency of lower risk of infection among young people. Older people have a higher cumulative exposure to the risks of becoming infected with *T. gondii*, since they have had more contact with the forms of transmission of the parasite [11, 39, 47–49]. This was not observed in individuals with heterozygous beta-thalassemia because there were no under 18-year-old individuals in the group or, for patients with homozygous beta-thalassemia, because the number of patients was too small.

Carriers of these hemoglobinopathies that are treated with transfusions should receive filtered packed red blood cells, that is, the leucocytes should be removed from whole blood during the fractionation process [27, 30, 50–52]. One should remember that, as a rule, *T. gondii* is an intracellular parasite that is found in peripheral leukocytes. Thus, the use of leukocyte filters may be an important factor contributing to a reduction in the transfusion-related transmission of toxoplasmosis [30, 32, 51].

However, this study did not evaluate the transfused blood components nor the filters discarded after transfusions, which may be an excellent way to evaluate *T. gondii* infection particularly as the method is non-invasive and the filters are thrown away.

## Conclusions

A comparison by age of patients with sickle cell anemia showed a trend of lower risk of infection among younger individuals. Patients with beta-thalassemia and sickle cell anemia in Brazil are infected by *T. gondii* as seen by the presence of anti-*T. gondii* IgM and IgG antibodies.

## Abbreviations

*T. gondii*: *Toxoplasma gondii*
IgG: immunoglobulin G
IgM: immunoglobulin M
PCR: polymerase chain reaction
ELISA: enzyme-linked immunosorbent assay
EDTA: ethylenediaminetetraacetic acid
